# Retraction Note: Supported black phosphorus nanosheets as hydrogen-evolving photocatalyst achieving 5.4% energy conversion efficiency at 353 K

**DOI:** 10.1038/s41467-023-38969-6

**Published:** 2023-06-22

**Authors:** Bin Tian, Bining Tian, Bethany Smith, M. C. Scott, Ruinian Hua, Qin Lei, Yue Tian

**Affiliations:** 1 Key Lab of Advanced Transducers and Intelligent Control System of Ministry of Education, College of Physics and Optoelectronics, Taiyuan University of Technology, Taiyuan, 030024 China; 2 Department of Materials Science and Engineering, University of California, Berkeley, Berkeley, CA 94720 USA; 3 Molecular Foundry, Lawrence Berkeley National Lab, Berkeley, CA 94720 USA; 4 College of Life science, Dalian Nationalities University, Dalian, 116600 China

Retraction to: *Nature Communications* 10.1038/s41467-018-03737-4, published online 11 April 2018

The authors have retracted this article following discussion with Editors. It has come to our attention that there has been incorrect use of data in some figures in the published paper. The figure panels affected are Figure 1d, Supplementary Figure 5, Supplementary Figure 18, and Supplementary Figure 20. Some data from our previously published work^1^ was inadvertently duplicated during the figure preparation for this paper. The figures in question are Figure 1b and 1c, Figure 3a and 3b, and Supplementary Figure 7. There was an error in the value of the x-axis in Figure 4b. Additionally, the original AFM image had not been processed properly thus the line-cut profiles presented (Figure 2e) are incorrect. We have shared the corrected Figures with the Editorial office, and all original data and images regarding the characterizations in question, including XRD, XPS, absorption spectra and AFM images, can be provided on request. In view of so many figures being affected, we wish to retract the Article. In addition, although Yue Tian worked at the Molecular Foundry, the affiliation should not be included for this study. All authors agree to this retraction.
